# Clinical and Expression Significance of AKT1 by Co-expression Network Analysis in Endometrial Cancer

**DOI:** 10.3389/fonc.2019.01147

**Published:** 2019-11-06

**Authors:** Xiao Huo, Hengzi Sun, Qian Liu, Xiangwen Ma, Peng Peng, Mei Yu, Ying Zhang, Dongyan Cao, Keng Shen

**Affiliations:** ^1^Department of Obstetrics and Gynecology, Peking Union Medical College Hospital, Chinese Academy of Medical Sciences and Peking Union Medical College, Beijing, China; ^2^Department of Obstetrics and Gynecology, Beijing Chao-Yang Hospital, Capital Medical University, Beijing, China

**Keywords:** endometrial cancer, AKT1, GEO, WGCNA, bioinformatics

## Abstract

**Introduction:** Endometrial cancer is one of the most common uterine cancers worldwide. AKT is reported to regulate progesterone receptor B dependent transcription and angiogenesis in endometrial cancer. However, the potential mechanisms of AKT in the tumor progression of endometrial cancer remain unclear.

**Methods:** We used GSE72708 with gene expression profiles of AKT regulation from the GEO database. We performed GSEA analysis to explore pathway enrichments. We found that most upregulated enriched pathways in siAKT group were associated with acid metabolism and immune network. Endometrial cancer and various signaling pathways were downregulated enriched. Moreover, different molecular mechanism of regulation between progestin (R5020) and AKT was identified, which were related to VEGF signaling pathway. The hub genes were evaluated by immunohistochemical staining of endometrial cancer tissues.

**Results:** We screened out a total of 623 differentially expressed genes among different groups. According to weighted gene co-expression network analysis (WGCNA) method, four distinct modules were identified. We found brown module showed a very high positive correlation with siAKT group and a very high negative correlation with R5020 group. A total of six hub genes including PBK, BIRC5, AURKA, GTSE1, KNSTRN, and PSMB10 were finally identified associated with AKT1. In addition, the data also shows that the higher expression of AKT1, GTSE1, BIRC5, AURKA, and KNSTRN is significantly associate with poor prognosis of endometrial cancer.

**Conclusion:** Our study identified six hub genes related to the prognosis of endometrial cancer, which may provide new insights into the underlying biological mechanisms driving the tumorigenesis of endometrial cancer, especially in AKT1 regulation.

## Introduction

Uterine cancer among women was frequently diagnosed worldwide. Endometrial cancer (EC) is the most common uterine cancer that is commonly diagnosed worldwide, accounting for ~382,069 new cases every year with an incidence rates of 8.4 per population (1). Moreover, the mortality rates were 4.3–4.5 and 8.2–8.9 per 100,000 population for the white and black race in Unite States, respectively, and 2.5 per 100,000 population for the yellow race in China (2, 3). With the advances in diagnosis and treatment strategies and techniques, the 5-year survival rate of localized endometrial cancer has achieved to 95% (3). While 80% of endometrial cancer is endometroid adenocarcinoma which is hormonally driven and hormone dependent (4). At present, nearly 5% of women with endometrial cancer are under age 40, these young patients usually have a long history of abnormal uterine bleeding or infertility and have strong desire to maintain their reproductive potential. High dose progesterone is the most commonly used fertility preservation option for stage 1, grade 1, endometrioid adenocarcinoma confined to endometrium (5). Moreover, hormone therapy also was recommended for the recurrent, metastatic or high-risk disease as systemic therapy (6). However, the response rates to progestin therapy vary and the underlying molecular mechanisms of progestin insensitivity are still poorly understood by people. Therefore, the identification of biological ways to improve the prognosis or explore significant molecular functions in endometrial cancer is necessary. This could help understand its pathogenesis and provide more effective treatment of endometrial cancer.

AKT is reported to regulate progesterone receptor B (PRB)-dependent transcription and angiogenesis in endometrial cancer (7). In addition, the phosphoinositide 3-kinase (PI3K)/AKT signaling pathway was activity in endometrial cancer, which can promote the proliferation of endometrial cancer cells (8). It was reported that it is the activation of AKT resulted to the proliferation and metastasis of endometrial stromal cells (9, 10). In human cancers, AKT1 plays a central role in tumorigenesis. Moreover, it is involved in various cellular processes, such as apoptosis, proliferation, cell metabolism, cell cycle, and transcription of protein (11). In endometrial cancer, the activation of AKT1 in adult endometrium was enough to initiate endometrial cancer (12). Therefore, the PI3K/AKT signaling pathway is the most commonly mutated pathway in type I endometrial cancers, which indicated that it played an important role in the pathogenesis of endometrial cancer (8).

Recently, with rapid development of the microarray and sequencing technology, as well as available open-access databases such as the Gene Expression Omnibus (GEO) and The Cancer Genome Atlas (TCGA), the identification of key genes in human cancers and discovery of molecular markers have been performed including endometrial cancer. The TCGA database integrates a large amount of clinical information and gene sequencing data, allowing for comprehensive analysis of various of cancers. The gene expression profiling interactive analysis (GEPIA), which is an web server for cancer gene expression profiling and interactive analyses (13), provides tumor/normal differential expression analysis, according to different cancer characteristic such as cancer types or pathological stages. Moreover, survival analysis, similar gene detection and gene expression-survival correlation analysis are also can be performed. Weighted gene co-expression network analysis (WGCNA) which is widely used to capture the hub gene according to explore the relationships of different genes by constructing free-scale gene co-expression networks. WGCNA has been widely used to capture the hub genes. For instance, Yin et al. (14) used the gene expression profiles of chromophobe renal cell carcinoma from TCGA database. A total of 2,215 differentially expressed genes (DEGs) were screened out, and eight co-expression modules were constructed by WGCNA method. The highest correlations with pathologic stage, neoplasm status, and survival status were identified as the key modules. Thus, 29 candidate hub genes were identified. In another study (15), microarray data of 101 clear cell renal cell carcinoma samples and 95 normal kidney samples were analyzed and 2,425 DEGs were screened. Eleven co-expressed gene modules were identified by WGCNA. In endometrial cancer, there was only one study using WGCNA method in 2014 (16).

In this study, by integrating different bioinformatic methods, and analyzing clinical information and gene expression profiles of endometrial cancer patients, we aim to investigate the potential mechanisms of AKT1 in the tumor progression of endometrial cancer. Our results may improve the understanding of pathogenesis of endometrial cancer. Moreover, it may provide insight regarding the novel treatment of endometrial cancer.

## Materials and Methods

### Gene Expression Datasets and Clinical Pathological Data

Microarray-based dataset GSE72708 (7), which contains a total of 12 Ishikawa endometrial cancer cells were obtained from the GEO database (https://www.ncbi.nlm.nih.gov/) and annotated according to the Illumina HumanHT-12 V4.0 platform. In the overall design of this dataset, Ishikawa endometrial cancer cells were transfected with either siCtrl or siAKT. Cells were then serum starved overnight and then treated with either Ethanol Vehicle Control (VC) or 10 nM progestin (R5020) for 24 h. Each treatment was performed in triplicate. When multiple probes corresponded to one specific gene, the median expression level was considered its final expression. The RNA sequencing data of endometrial cancer obtained from TCGA dataset (https://cancergenome.nih.gov/), were used as an independent validation cohort. In this study, we enrolled patients histologically diagnosed as endometrial cancer for further analysis.

### Gene Set Enrichment Analysis (GSEA)

In order to explore biological pathways of different groups, GSEA was used. The annotated gene sets of c2.cp.kegg.v6.1.symbols.gmt were considered as the reference gene sets. The number of permutations was 1,000. Other parameters were set to default. Significant differences at *p*-value < 0.05 was defined as the cutoff criteria. The EnrichmentMap plug-in of the Cytoscape software (version 3.6.1) was used to integrate and visualize these pathways.

### Identification of DEGs

Open-source software R language (Version 3.3.3, https://www.r-project.org/) and a R package of Bioconductor (http://www.bioconductor.org/) *limma* (17) package were utilized to identified DEGs between progestin (R5020), siAKT, and R5020+siAKT groups. A |Fold Change (FC)| > 1.3 and false discovery rate (FDR) < 0.05 were set as cut-off criteria for the screening of DEGs. Differentially expressed genes were selected for co-expression analysis. The Volcano plot and heatmap were generated by *base* R package. The results of gene intersections were used *UpSetR* R package.

### Functional Enrichment Analysis of DEGs

In order to explore the potential mechanism, we performed KEGG pathway enrichment analysis by *clusterprofiler* (18) package in R software (Version 3.3.3). FDR < 0.1 was set as cut-off value. The *clusterProfiler* is an ontology-based R package that not only automates the process of biological-term classification and the enrichment analysis of gene clusters, but also provides a visualization module for displaying analysis results.

### Construction of Gene Co-expression Network by WGCNA Method

Scale-free gene co-expression networks were constructed by the *WGCNA* package (19). To ensure that the results of network construction were reliable, outlier samples were removed. An appropriate soft threshold power was selected in accordance with standard scale-free networks, with which adjacencies between all differentially expressed genes were calculated by a power function. Then, the adjacency was transformed into a topological overlap matrix (TOM), and the corresponding dissimilarity (1-TOM) was calculated. Module identification was accomplished with the dynamic tree cut method by hierarchically clustering genes using 1-TOM as the distance measure with a deepSplit value of two and a minimum size cutoff of 30 for the resulting dendrogram. Highly similar modules were identified by clustering and then merged together with a height cut-off of 0.25.

### Functional Enrichment Analysis

To explore biological functions of above significant genes, all genes in brown and yellow module were mapped into the gprofiler (http://biit.cs.ut.ee/gprofiler) (20).

### Identification of Hub-Gene

We uploaded all genes in the brown module into the Search Tool for the Retrieval of Interacting Genes (STRING) database (21) to build the protein-protein interaction (PPI) network. The hub genes in the module were defined by using network construction and those genes choosing a confidence > 0.4 to construct a PPI. In the PPI network, genes with a connectivity degree ≥ 4 (node/edge) were defined hub genes and used for further analysis.

To explore the expression patterns between tumor and normal tissues of endometrial cancer, GEPIA database (http://gepia.cancer-pku.cn) (13) was used. This database is an interactive web server for analyzing the RNA sequencing expression data from the TCGA projects. The gene expression profiles of paired tumor and normal tissues were used. In the validation analysis, the gene expression of samples lower than 25% of total samples were considered as low-AKT1 group. The gene expression of samples higher than 75% of total samples were considered as high-AKT1 group.

### Immunohistochemical Staining (IHC)

We collected a total of 182 progesterone receptor positive human endometrial tissue samples, 107 stage III-IV cancer tissue of which had accompanying follow-up information, and 75 cancer-adjacent endometrial tissue samples from archives of paraffin-embedded tissues between May, 2011 and May, 2014 at the Department of Pathology of Peking Union Medical College Hospital. The follow-up was performed until May 30, 2019. The pathological diagnoses were reconfirmed by a pathologist. The project was approved by the Ethical Committee (Peking Union Medical College Hospital), and informed consent was acquired from patients or family members. IHC was performed as previously described (22). Anti-antibody (AKT1 1:250, Abcam, ab235958; PR 1:100, Abcam, ab32085; PBK 1:100, Abcam, ab75987; BIRC5 1:800, Abcam, ab469; AURKA 1:100, Abcam, ab52973; GTSE1 1:50, Abcam, ab103232; KNSTRN 1:50, Abcam, ab122769; PSMB10 1:100, Abcam, ab183506) was used for IHC. The scoring details have been described previously (23).

## Results

### Data Processing and GSEA Analysis

In this study, we performed a multi-steps analysis to explore the molecular mechanisms of AKT1 in the pathogenesis of endometrial cancer. First, we selected the GSE72708 dataset with gene expression profiles from GEO database, which contained 12 PRB-Ishikawa samples, including a total of four different groups. They were siCtrl or siAKT groups and treated with either Ethanol Vehicle Control (VC) or progestin (R5020) for 24 h.

According to the probe annotation information, we then map the probes to gene symbols. Next, the gene expression profiles were used quantile normalization and when multiple probes corresponded to one specific gene, the median expression level was considered its final expression.

**Figure 1:**
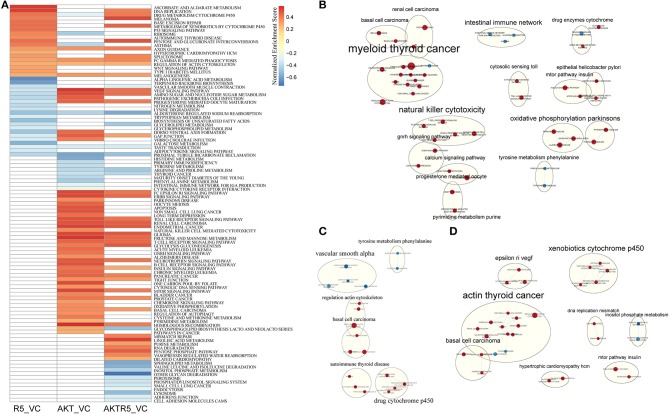
The results of GSEA analysis. **(A)** The heatmap of GSEA enriched pathways among different treatment groups. **(B)** The pathway network of siAKT group. **(C)** The pathway network of R5020 group. **(D)** The pathway network of siAKT+R5020 group. The blue dots represent upregulated pathways and the red dots were downregulated pathways.

To compare the different effects of R5020 and siAKT on the biological pathways, we used GSEA analysis (Supplementary S1). As shown in Figure 1A, there were total of 40 pathways in R5020 groups (vs. control groups), and 64 enriched pathways in siAKT groups (vs. control groups). There were 17 mutual pathways between these two groups, such as regulation of actin cytoskeleton, VEGF signaling pathway, and GAP junction. In addition, there were 57 enriched pathways in siAKT+R5020 group. Moreover, we found that most upregulated enriched pathways in siAKT group were associated with acid metabolism and immune network. Endometrial cancer and various signaling pathways were downregulated enriched (Figure 1B). The results of R5020 group were associated with acid metabolism. Downregulated pathways were enriched in DNA activities and signaling pathway (Figure 1C). Interestingly, the VEGF signaling pathway was upregulated in R5020 group, however, downregulated in siAKT group. As shown in Figure 1D, VEGF signaling pathway was also downregulated in siAKT+R5020 group. Above results suggested that different molecular mechanism of regulation between R5020 and AKT, which were related to VEGF signaling pathway.

### Identification of DEGs Among Different Treatment Groups

**Figure 2:**
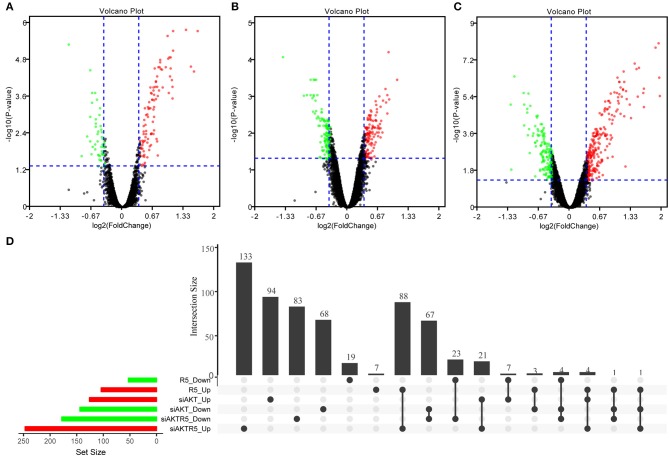
The results of DEGs among different groups. **(A)** The volcano plot of DEGs in R5020 group. **(B)** The volcano plot of DEGs in siAKT group. **(C)** The volcano plot of DEGs in siAKT+R5020 group. **(D)**The gene intersections among different groups.

In order to observe the effects of R5020, siAKT, and R5020 + siAKT on gene expression, we used R software package *limma* package to screen out the genes differentially expressed among different groups (Supplementary S2). The difference results of each comparison group were show in Supplementary S3. In brief, we found a total of 157 significant DEGs in R5020 group, including 104 up- and 53 downregulated DEGs. Moreover, there were 270 DEGs in siAKT group, including 126 up- and 144 downregulated DEGs. The volcano plot was shown in Figures 2A–C and the results of gene intersections were shown in Figure 2D. We found that a small number of intersected genes between R5020 and siAKT groups, suggesting that the treatments of R5020 and siAKT affected the expression of genes and thus played different roles in biological regulations.

### Functional Enrichment Analysis of DEGs

To investigate the molecular mechanism of those genes, we performed functional enrichment analysis. The DEGs were processed by KEGG pathway enrichment analysis (Supplementary S4). As shown in Supplementary S5, we found that upregulated DEGs of R5020 group were significantly enriched in mineral absorption, fructose and mannose metabolism, and aldosterone-regulated sodium reabsorption. Downregulated DEGs were enriched in cytokine-cytokine receptor interaction. In addition, upregulated genes of siAKT group were also enriched in mineral absorption. Downregulated genes of siAKT group were enriched in a total of 18 various cancer pathways, such as central carbon metabolism in cancer, apoptosis, and mTOR signaling pathway. Above results suggested that the tumor inhibition effect of siAKT treatment was more obvious than R5020 treatment.

### Construction of Co-expression Modules by WGCNA Method

**Figure 3:**
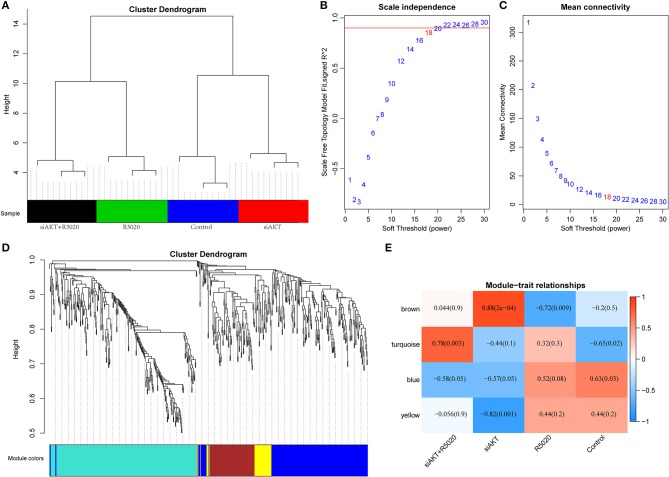
Construction of co-expression modules by WGCNA method. **(A)** The results of clustering analysis. **(B)** Analysis of the scale-free fit index for various soft-thresholding powers (β). **(C)** Analysis of the mean connectivity for various soft-thresholding powers. **(D)** Dendrogram of all DEGs clustered based on a dissimilarity measure (1-TOM). **(E)** Heatmap of the correlation between module eigengenes and sample groups of endometrial cancer.

All DEGs that we obtained were included for constructing co-expression modules by WGCNA algorithm. First, 623 DEGs were used in clustering analysis that they can clearly distinguish the four types of samples (Figure 3A). Next, we used 623 DEGs to establish a co-expression network. First of all, the appropriate power value was screened out. When the power value was equal to 18, scale free networks were constructed with best topology module fit index and the relatively higher average connectivity (Figures 3B,C). Therefore, four distinct gene co-expression modules were identified by the appropriate power value (Figure 3D, Supplementary S6). There was a close internal similarity in each module. By computing the Pearson correlation coefficients between ME of each module and each group of samples (Figure 3E), which means that the higher the correlation coefficient, the closer of the relationship is between the module and the group of samples. From the graph, we can see that the brown module showed a very high positive correlation with siAKT group and a very high negative correlation with R5020 group. Moreover, yellow module was significantly negatively correlated with siAKT group. Above results suggested that genes in brown module may cause the difference between R5020 and siAKT treatments. And the genes in the yellow module are co-expressed with AKT.

### Functional Enrichment Analysis of Modules

To explore biological functions of these genes in above two modules, all the genes in brown and yellow modules were mapped into g:profiler (Supplementary S7). As the results of brown module, we found that genes were enriched in GO terms and signaling pathways. In addition, genes in yellow module were enriched in Protein databases such as Human Protein Atlas (HPA). As shown in Supplementary S8A, genes in brown module were mainly enriched in immune response and cell death, which played important roles in the development of tumor progression. However, genes in yellow module were not enriched in pathways. They were only enriched in tissues (Supplementary S8B).

### Identification of Hub Genes Associated With AKT1

**Figure 4:**
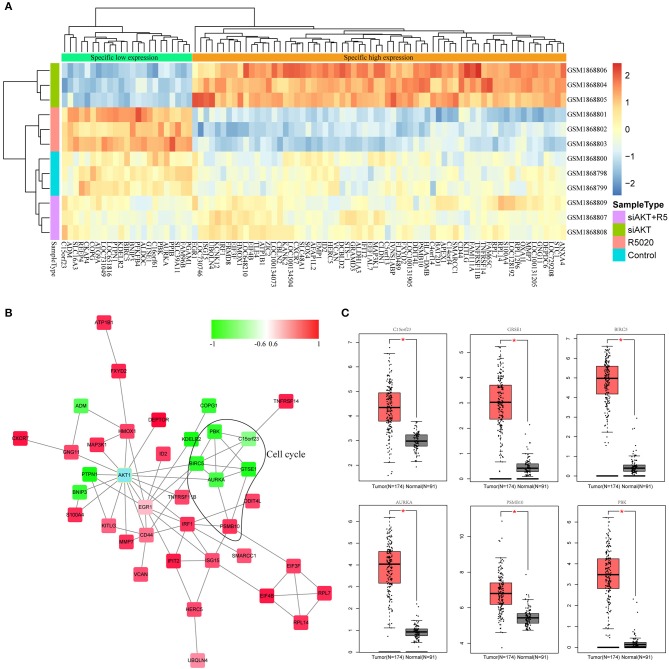
Identification of hub genes associated with AKT. **(A)** The results of clustering analysis based on genes in brown module. **(B)** The PPI network of genes in brown module. **(C)** The box plot of differentially expressed patterns of six hub genes in endometrial cancer.

In order to select genes associated with AKT1, we used the gene expression profile in the brown module and performed hierarchical clustering analysis to observe the expression patterns of these genes in each sample. As shown in Figure 4A, these genes could clearly divide all the samples into two group. Then, all of these genes were subjected to STRING database and the interactions between these genes with AKT1 were obtained. We selected the genes with the shortest path to AKT1 as the final interaction network by Cytoscope for visualization (Figure 4B). The shortest path between gene and AKT1 is <2. Furthermore, the plug-in of *ClusterOne* in Cytoscape was used to search the hub genes. A total of six genes including PBK, BIRC5, AURKA, GTSE1, KNSTRN(C15orf23), and PSMB10 were finally identified, among them, five are negatively correlated with the brown module.

Next, we applied GEPIA (http://gepia.cancer-pku.cn) to detect the difference in expression levels of the hub genes between tumor and normal tissues (Figure 4C). The results indicated that all these six hub genes were significantly differentially expressed in endometrial cancer.

### The Expression and Clinical Significance of Hub Genes

**Figure 5:**
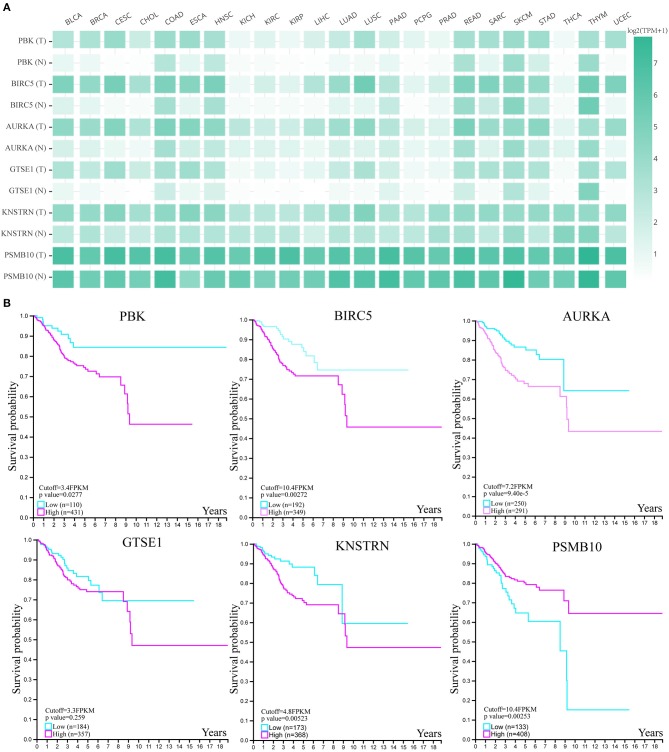
The expression and clinical significance of hub genes. **(A)** The expression heatmap of six genes in human cancers. **(B)** The survival K-M plot of six genes.

In order to explore the expression profiles of these six hub genes in other cancers, we used GEPIA database. As shown in Figure 5A, we found that above six genes were all expressed in other tumor tissues, however, lower expressed in paired normal tissues. Next, we used HPA database to investigate their clinical significances (Figure 5B). The K-M plots showed that, except GTSE1, all genes were significantly associated with endometrial cancer patients' prognosis. Above results suggested that these genes may have great impact on the progression and development of endometrial cancer.

**Figure 6:**
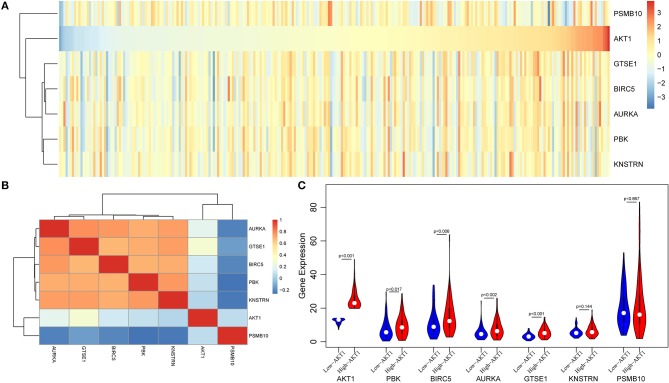
The validation analysis of AKT in TCGA database. **(A)** The expression heatmap of six genes and AKT in TCGA database. **(B)** The Pearson correlation coefficient of these genes and AKT. **(C)** The violin plot of these six genes in high-AKT and low-AKT groups.

Then, we used the gene expression profiles from TCGA database to valid our results. A total of 578 endometrial cancer patients, including 543 tumors and 35 adjacent normal tissues were obtained from TCGA database. First, we selected a total of 258 patients with stable expression of PGR gene as endometrial cancer patients, whose expressions were higher than half of samples. Then, we performed hierarchical cluster and correlation analyses. As shown in Figure 6A, there were close corrections among five genes including GTSE1, BIRC5, AURKA, PBK, and KNSTRN. When calculating the Pearson correlation coefficient of these genes, we found that all above five genes were positive corrections with AKT1, however, PSMB10 presented a negative correction with AKT1 (Figure 6B). Furthermore, four of these six genes were differentially expressed between high-AKT1 group and low-AKT1 group (Figure 6C). The remaining two genes, KNSTRN and PSMB10, were also notably differentially expressed, although the difference was not statistically significant.

### Evaluation of the Hub Genes by IHC

From May, 2011 to May, 2014, a total of 182 human endometrial tissue samples, 107 stage III-IV cancer of which had accompanying follow-up information, and 75 cancer-adjacent endometrial tissue samples from archives of paraffin-embedded tissues was collected at the Department of Pathology of Peking Union Medical College Hospital. The follow-up was performed until May 30, 2019. Supplementary S9 summarizes the characteristic of all patients, including, age, disease stage, and tumor grade. We selected the six hub genes (GTSE1, BIRC5, AURKA, PBK, KNSTRN, and PSMB10) and AKT1 to evaluate gene expression values in endometrial cancer using IHC.

**Figure 7:**
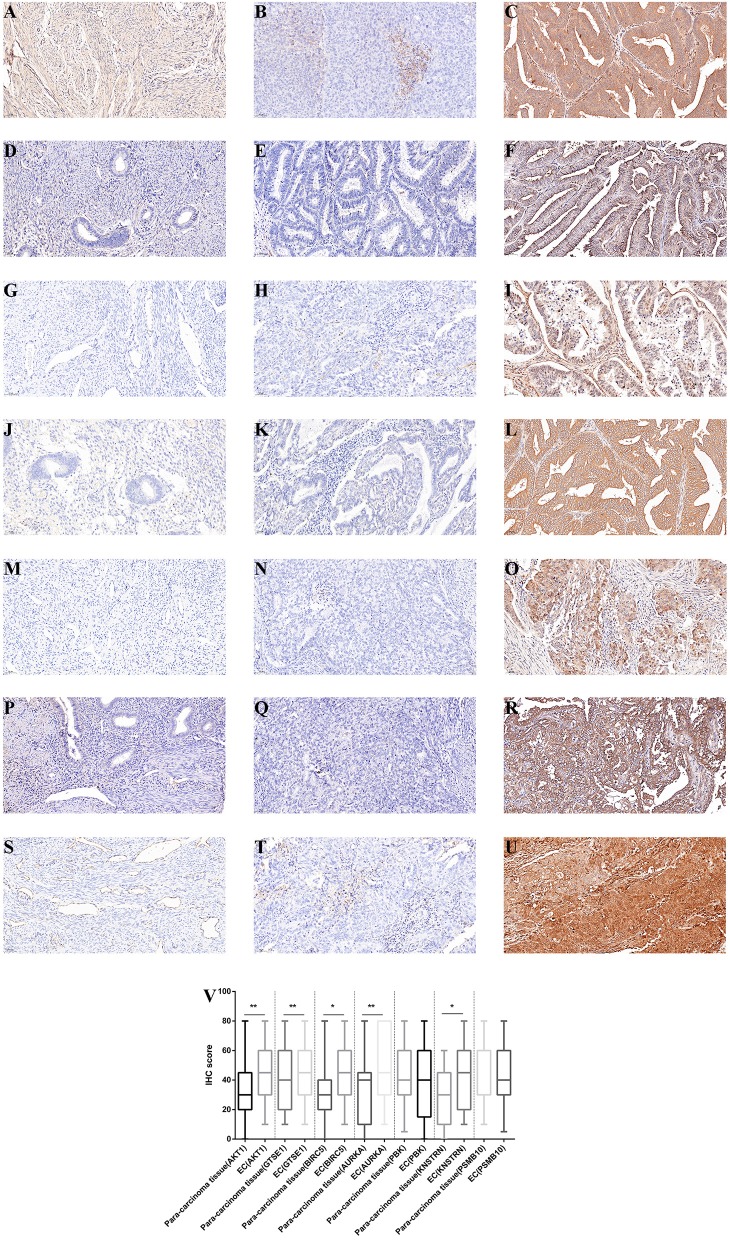
Immunohistochemistry for AKT1, GTSE1, BIRC5, AURKA, PBK, KNSTRN, and PSMB10. Samples from cancer-adjacent endometrial tissue (*N* = 75) and endometrial cancer (*N* = 107). Cancer-adjacent endometrial tissue sample of weak immunostaining score for either AKT1 **(A)**, GTSE1 **(D)**, BIRC5 **(G)**, AURKA **(J)**, PBK **(M)**, KNSTRN **(P)**, and PSMB10 **(S)**. Endometrial cancer sample of weak and strong immunostaining score for either AKT1 **(B,C)**, GTSE1 **(E,F)**, BIRC5 **(H,I)**, AURKA **(K,L)**, PBK **(N,O)**, KNSTRN **(Q,R)**, and PSMB10 **(T,U)**. The expression for each gene were depicted in **(V)** slides (200 X, ^*^p < 0.05, ^**^p < 0.01).

**Figure 8:**
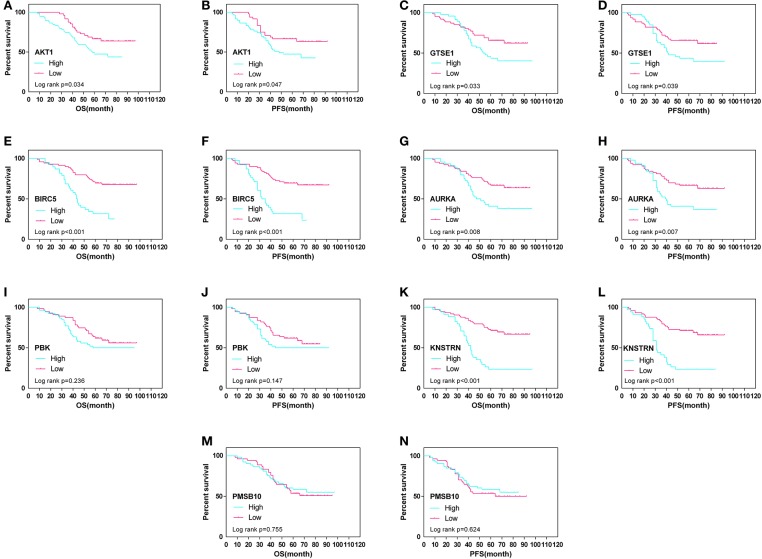
Overall (OS) and progression-free survival (PFS) curves in endometrial cancer (*N* = 107) according to AKT1 **(A,B)**, GTSE1 **(C,D)**, BIRC5 **(E,F)**, AURKA **(G,H)**, PBK **(I,J)**, KNSTRN **(K,L)**, and PSMB10 **(M,N)** genes expression status.

The expression differences of GTSE1, BIRC5, AURKA, PBK, KNSTRN, PSMB10, and AKT1 between endometrial cancer tissues and adjacent normal endometrial tissues were explored, as shown in Figure 7. The correlation of the expression of AKT1 and GTSE1, BIRC5, AURKA, PBK, KNSTRN, PSMB10 is shown in Table 1. The data shows that the expression of BIRC5 (*p* = 0.0035) and KNSTRN (*p* = 0.0002) was significantly correlation to the expression of AKT1. The expression of GTSE1 (*p* = 0.1312) and AURKA (*p* = 0.075) has not got the statistics significant correlation to the expression of AKT1, which might due to the insufficient cases in present study. The AKT1 (49.53 ± 1.965 vs. 34.40 ± 2.358, *p* < 0.01), GTSE1 (45.23 ± 2.045 vs. 37.33 ± 2.182, *p* = 0.01), BIRC5 (47.85 ± 2.197 vs. 27.80 ± 2.003, *p* < 0.01), AURKA (49.95 ± 2.408 vs. 34.40 ± 2.603, *p* < 0.01) and KNSTRN (43.79 ± 2.357 vs. 30.07 ± 2.039, *p* < 0.01) shows significantly higher expression in endometrial cancer than cancer adjacent tissue. In addition, the correlation between the expression of these genes and the prognosis of endometrial cancer is shown in Figure 8. These data show that the higher expression of AKT1 (OS, HR = 1.863, 95% CI 1.050-3.218, *p* = 0.034; PFS, HR = 1.789, 95% CI 1.014-3.107, *p* = 0.047), GTSE1 (OS, HR = 1.817, 95% CI 1.056–3.306, *p* = 0.072; PFS, HR = 1.782, 95% CI 1.038–3.245, *p* = 0.039), BIRC5 (OS, HR = 3.130, 95% CI 2.109–7.325, *p* < 0.001; PFS, HR = 3.192, 95% CI 2.203–7.691, *p* < 0.001), AURKA (OS, HR = 2.082, 95% CI 1.230–3.920, *p* = 0.008; PFS, HR = 2.097, 95% CI 1.249–3.987, *p* = 0.008), and KNSTRN (OS, HR = 3.619, 95% CI 2.742–10.21, *p* < 0.001; PFS, HR = 3.563, 95% CI 2.705–10.02, *p* < 0.001) were associated with poor prognosis in endometrial cancer patients. In addition, the higher expression of PBK (OS, HR = 1.398, 95% CI 0.802–2.474, *p* < 0.236; PFS, HR = 1.386, 95% CI 0.797–2.456, *p* < 0.147) was also associated with poor prognosis, though it didn't shown significance in present IHC evaluation.

**Table 1:** The cases of different gene expression in endometrial cancer.

**Gene**	**GTSE1 (+)**	**GTSE1 (−)**	**BIRC5 (+)**	**BIRC5 (−)**	**AURKA (+)**	**AURKA (−)**	**PBK (+)**	**PBK (−)**	**KNSTRN (+)**	**KNSTRN (−)**	**PSMB10 (+)**	**PSMB10 (−)**
AKT1 (+)	32	27	40	19	36	23	35	24	41	18	40	19
AKT1 (−)	19	29	19	29	21	27	28	20	16	32	34	14
χ^2^	0.1312		0.0035		0.075		0.9177		0.0002		0.7879	

*+ High expression, − Low expression*.

## Discussion

The PI3K pathway regulates key biology aspects of cancer including metabolism, cellular growth, and survival (24). After be recruited to the membrane by PI3K phosphorylated, AKT indirectly inhibits mTOR complex 1 (mTORC1) which is a key regulator of metabolism and biosynthetic processes (25, 26). Therefore, the PI3K/AKT/mTOR pathway plays an important role in gynecological carcinogenesis. Meanwhile, the PI3K-Akt signal pathway is also the most frequently mutated pathway in type I endometrial cancer. Most type I endometrial tumors had more or less somatic alterations related to this pathway (27). Siming Zhao et al. also reported that a significantly increased burden of mutation in 14 genes, including PIK3CA, TP53, PPP2R1A, KRAS, FBXW7, CHD4, TAF1, PTEN, HCFC1R1, CDKN1A, CTDSPL, YIPF3, SPOP, and FAM132A, in the whole-exome sequencing of 57 uterine serous carcinoma compared to their matched normal DNA from the same patients (28). Recently, studies have showed that AKT can regulate the PR-dependent transcription and angiogenesis, and progestin resistance can be reversed by inhibiting the PI3K/AKT pathway in endometrial cancer (7, 29). Actually, our previous study which focused on AKT also find that siAKT significantly inhibit the proliferation of PR positive endometrial cancer cells and progesterone resistance endometrial cancer cells than progestin treatment. However, the molecular mechanism and more specific therapy target is still unclear.

Recently, with the help of next generation sequencing technology, many gene expression studies on human cancer have been reported on the public databases in the last decade. In endometrial cancer, Xu et al. (30) performed a lncRNA-based signature risk score to predict patient's survival by the genome-wide analysis of the lncRNA expression profiling in TCGA. In another study, a six-gene prognostic signature, including CTSW, PCSK4, LRRC8D, TNFRSF18, IHH, and CDKN2A, in endometrial cancer was also established using robust likelihood-based survival modeling (31). Meanwhile, Ying et al. (32) elevated the mortality risk of endometrial cancer patients according to a novel prognostic model which was performed using stepwise selection of endometrial cancer TCGA database. However, most studies focused on prognostic gene panels of endometrial cancer.

In present study, we have performed a multi-steps bioinformatics analysis in endometrial cancer using the gene expression profiles from GEO database and identified a set of genes that may be regulated by AKT1. We expected to explore the DEGs between siAKT and progesterone treatment by bioinformatics, and to evaluate these DEGs in PR-positive endometrial carcinoma tissues with prognostic information, so as to identify the more specific therapeutic targets, which may improve the clinical treatment response for endometrial cancer patients.

The present study identified six hub genes in the networks, including PSMB10, GTSE1, BIRC5, AURKA, PBK, and KNSTRN. In agreement with previous study, these hub genes were differentially expressed and significantly correlated with patient's survival in a variety of human tumors. For example, PSMB10 (Proteasome subunit beta type-10), as part of an immunoproteasome, has a major role in the immune system. In various type of human malignancies, the overexpression of GTSE1 (G-2 and S-phase expressed 1) has been reported as a potential marker associated to metastasis (33). The paclitaxel sensitivity of cancer cells and cisplatin-induced cell death was enhanced after silencing the expression of GTSE1. Moreover, study also demonstrated that it diminishes the defects of chromosome segregation in highly CIN cancer cell lines (34). As a target gene of the TEAD4 transcription factor, GTSE1, which transcriptional regulation related to the YAP and TAZ coactivator, plays an important role in triple-negative breast cancer (35). In addition, it was found as hub gene in kidney renal clear cell carcinoma (36). BIRC5 (baculoviral IAP repeat containing five) is a member of the inhibitor of apoptosis (IAP) gene family, which can prevent apoptotic cell death by encoding negative regulatory proteins. Previous study has reported that overexpression of PI3K can induce the expression of BIRC5 mRNA through Akt activation. Besides, it also indicated that p70S6K1 is an important downstream of PI3K and Akt in regulating the expression of BIRC5 (37). Recently, Chuwa et al. have demonstrated that overexpression of BIRC5 was an independent prognostic factor for poor progression free survival of endometrial cancer patients. Moreover, knockdown of BIRC5 in endometrial cancer cell lines induced cell apoptosis, which indicated that BIRC5 is not only a prognostic factor also a promising therapeutic target for endometrial cancer (38). AURKA (aurora kinase A) was shown to promote cell proliferation and predicts poor prognosis in bladder cancer (39). Moreover, based on bioinformatics analysis, this gene was also considered as hub gene in bladder cancer (40). AURKA also was reported to be associated with poor prognosis of smoking related lung adenocarcinoma using bioinformatics analysis (41). Overexpression of PBK (PDZ binding kinase) has been implicated in tumorigenesis. It was also reported as hub gene in diffuse large B-cell lymphoma (42). Besides, PBK also played important roles in other cancers, including lung adenocarcinoma (43, 44). Moreover, KNSTRN [Kinetochore Localized Astrin (SPAG5) Binding Protein], which was essential for the mitotic spindle, faithful chromosome segregation and progression into anaphase, was reported to be involved in pathogenesis of leukemias (45), basal cell carcinoma (46). Moreover, Lee et al. performed the whole-exome sequencing on cutaneous squamous cell carcinoma samples and patients-matched normal skin samples to investigate the genetic causes of cutaneous squamous cell carcinoma. The results showed that KNSTRN was one of the top three most frequently mutated genes, which provided evidence that KNSTRN is an oncogene we were unfamiliar with. They search of publicly available TCGA data suggests KNSTRN might also play a role in melanoma. However, the role of KNSTRN in endometrial cancer was rarely be reported.

In present study, the IHC results also demonstrated that the expression of GTSE1, BIRC5, AURKA, PBK, and KNSTRN were positive corrections with AKT1. Moreover, the data also shows that the higher expression of AKT1, GTSE1, BIRC5, AURKA, and KNSTRN is significantly associate with poor prognosis of endometrial cancer. Therefore, these hub genes associated AKT1 may serve as important roles in the pathogenesis of endometrial cancer. Although some hub genes might not be the most specific markers for endometrial cancer, our study found that the part of them have a significant correlation with the prognosis of PR-positive endometrial cancer patients. Just as the BRCA genes play an important role in both breast and ovarian cancer, the present study also expected to identify new therapeutic targets for PR-positive endometrial cancer, although it might also play an important role in other cancers. Our results may provide new insights into the underlying biological mechanisms driving the tumorigenesis of endometrial cancer. However, present study lacks experimental studies to explore the molecular mechanism of these hub genes in endometrial cancer, which we will perform in near future. Moreover, we will also validate the prognostic value of the hub genes in more large population so that to evaluated the clinical benefit possibility.

## Conclusion

In conclusion, after a comprehensive analysis of the data of endometrial cancer patients, we have identified a set of hub genes that were regulated by AKT1 and may serve as potential biomarkers for prognosis prediction of endometrial cancer patients. This study highlights a novel understanding of the potential molecular mechanism in endometrial cancers tumorigenesis and provides novel molecular targets for the development of more effective therapies for the treatment of endometrial cancer.

## Data Availability Statement

Publicly available datasets were analyzed in this study. This data can be found here: https://www.ncbi.nlm.nih.gov/geo/query/acc.cgi?acc=GSE72708.

## Ethics Statement

All procedures performed in studies involving human participants were in accordance with the ethical standards of the institutional and national research committee and with the 1964 Helsinki declaration and its later amendments (The name and affiliation of the ethics committee that approved this study: The institutional ethics committee of Peking Union Medical College Hospital, CAMS Chinese Academy of Medical Sciences, No. S-326 2019). Written informed consent was obtained from all participants included in the study.

## Author Contributions

XH: study design and data analysis. HS: data collection, data analysis, manuscript writing, and follow up. DC and KS: funding and clinical data provider. QL, XM, PP, MY, and YZ: clinical data provider. All authors have read, edited, and approved of the final version of the manuscript.

### Conflict of Interest

The authors declare that the research was conducted in the absence of any commercial or financial relationships that could be construed as a potential conflict of interest.

## References

[1] Bray F Ferlay J Soerjomataram I Siegel RL Torre LA Jemal A. Global cancer statistics 2018: GLOBOCAN estimates of incidence and mortality worldwide for 36 cancers in 185 countries. CA Cancer J Clin. (2018) 68:394–424. 10.3322/caac.21492 30207593

[2] Chen W Sun K Zheng R Zeng H Zhang S Xia C Cancer incidence and mortality in China, 2014. Chin J Cancer Res. (2018) 30:1–12. 10.21147/j.issn.1000-9604.2018.01.01 29545714 PMC5842223

[3] Siegel RL Miller KD Jemal A Cancer statistics, 2019. CA Cancer J Clin. (2019) 69:7–34. 10.3322/caac.21551 30620402

[4] Rutgers JK. Update on pathology, staging and molecular pathology of endometrial (uterine corpus) adenocarcinoma. Future Oncol. (2015) 11:3207–18. 10.2217/fon.15.262 26551559

[5] van Weelden WJ Massuger L Pijnenborg JMA Romano A. Anti-estrogen treatment in endometrial cancer: a systematic review. Front Oncol. (2019) 9:359. 10.3389/fonc.2019.00359 31134155 PMC6513972

[6] Koh WJ Abu-Rustum NR Bean S Bradley K Campos SM Cho KR. Uterine neoplasms, version 1.2018, NCCN clinical practice guidelines in oncology. J Natl Comp Cancer Netw. (2018) 16:170–99. 10.6004/jnccn.2018.0006 29439178

[7] Lee II Maniar K Lydon JP Kim JJ. Akt regulates progesterone receptor B-dependent transcription and angiogenesis in endometrial cancer cells. Oncogene. (2016) 35:5191–201. 10.1038/onc.2016.56 26996671 PMC5031502

[8] Lee II Kim JJ. Influence of AKT on progesterone action in endometrial diseases. Biol Reprod. (2014) 91:63. 10.1095/biolreprod.114.119255 25100707 PMC4435059

[9] Kyo S Sakaguchi J Kiyono T Shimizu Y Maida Y Mizumoto Y. Forkhead transcription factor FOXO1 is a direct target of progestin to inhibit endometrial epithelial cell growth. Clin Cancer Res. (2011) 17:525–37. 10.1158/1078-0432.CCR-10-1287 21131554

[10] Wang X Li Y Wan L Liu Y Sun Y Liu Y. Downregulation of PDCD4 induced by progesterone is mediated by the PI3K/AKT signaling pathway in human endometrial cancer cells. Oncol Rep. (2019) 42:849–56. 10.3892/or.2019.7202 31233196

[11] Manning BD Cantley LC. AKT/PKB signaling: navigating downstream. Cell. (2007) 129:1261–74. 10.1016/j.cell.2007.06.009 17604717 PMC2756685

[12] Memarzadeh S Zong Y Janzen DM Goldstein AS Cheng D Kurita T. Cell-autonomous activation of the PI3-kinase pathway initiates endometrial cancer from adult uterine epithelium. Proc Natl Acad Sci USA. (2010) 107:17298–303. 10.1073/pnas.1012548107 20855612 PMC2951427

[13] Tang Z Li C Kang B Gao G Li C Zhang Z. GEPIA: a web server for cancer and normal gene expression profiling and interactive analyses. Nucleic Acids Res. (2017) 45:W98–102. 10.1093/nar/gkx247 28407145 PMC5570223

[14] Yin X Wang J Zhang J. Identification of biomarkers of chromophobe renal cell carcinoma by weighted gene co-expression network analysis. Cancer Cell Int. (2018) 18:206. 10.1186/s12935-018-0703-z 30564062 PMC6296159

[15] Chen L Yuan L Qian K Qian G Zhu Y Wu CL. Identification of biomarkers associated with pathological stage and prognosis of clear cell renal cell carcinoma by co-expression network analysis. Front Physiol. (2018) 9:399. 10.3389/fphys.2018.00399 29720944 PMC5915556

[16] Chou WC Cheng AL Brotto M Chuang CY. Visual gene-network analysis reveals the cancer gene co-expression in human endometrial cancer. BMC Genomics. (2014) 15:300. 10.1186/1471-2164-15-300 24758163 PMC4234489

[17] Ritchie ME Phipson B Wu D Hu Y Law CW Shi W. limma powers differential expression analyses for RNA-sequencing and microarray studies. Nucleic Acids Res. (2015) 43:e47. 10.1093/nar/gkv007 25605792 PMC4402510

[18] Yu G Wang LG Han Y He QY. clusterProfiler: an R package for comparing biological themes among gene clusters. Omics. (2012) 16:284–7. 10.1089/omi.2011.0118 22455463 PMC3339379

[19] Zhang B Horvath S. A general framework for weighted gene co-expression network analysis. Stat Appl Genet Mol Biol. (2005) 4:Article17. 10.2202/1544-6115.1128 16646834

[20] Reimand J Arak T Adler P Kolberg L Reisberg S Peterson H. g:Profiler-a web server for functional interpretation of gene lists (2016 update). Nucleic Acids Res. (2016) 44:W83–9. 10.1093/nar/gkw199 27098042 PMC4987867

[21] Szklarczyk D Morris JH Cook H Kuhn M Wyder S Simonovic M. The STRING database in 2017: quality-controlled protein-protein association networks, made broadly accessible. Nucleic Acids Res. (2017) 45:D362–8. 10.1093/nar/gkw937 27924014 PMC5210637

[22] Li YL Ye F Cheng XD Hu Y Zhou CY Lue WG. Identification of glia maturation factor beta as an independent prognostic predictor for serous ovarian cancer. Eur J Cancer. (2010) 46:2104–18. 10.1016/j.ejca.2010.04.015 20547056

[23] Zhang S-F Wang X-Y Fu Z-Q Peng Q-H Zhang J-Y Ye F. TXNDC17 promotes paclitaxel resistance via inducing autophagy in ovarian cancer. Autophagy. (2015) 11:225–38. 10.1080/15548627.2014.998931 25607466 PMC4502659

[24] Engelman JA Luo J Cantley LC. The evolution of phosphatidylinositol 3-kinases as regulators of growth and metabolism. Nat Rev Genet. (2006) 7:606–19. 10.1038/nrg1879 16847462

[25] Pearce LR Komander D Alessi DR. The nuts and bolts of AGC protein kinases. Nat Rev Mol Cell Biol. (2010) 11:9–22. 10.1038/nrm2822 20027184

[26] Ma XM Blenis J. Molecular mechanisms of mTOR-mediated translational control. Nat Rev Mol Cell Biol. (2009) 10:307–18. 10.1038/nrm2672 19339977

[27] Cheung LW Hennessy BT Li J Yu S Myers AP Djordjevic B. High frequency of PIK3R1 and PIK3R2 mutations in endometrial cancer elucidates a novel mechanism for regulation of PTEN protein stability. Cancer Discov. (2011) 1:170–85. 10.1158/2159-8290.CD-11-0039 21984976 PMC3187555

[28] Zhao S Choi M Overton JD Bellone S Roque DM Cocco E. Landscape of somatic single-nucleotide and copy-number mutations in uterine serous carcinoma. Proc Natl Acad Sci USA. (2013) 110:2916–21. 10.1073/pnas.1222577110 23359684 PMC3581983

[29] Gu C Zhang Z Yu Y Liu Y Zhao F Yin L. Inhibiting the PI3K/Akt pathway reversed progestin resistance in endometrial cancer. Cancer Sci. (2011) 102:557–64. 10.1111/j.1349-7006.2010.01829.x 21205080 PMC11159613

[30] Xu Q Yang Q Zhou Y Yang B Jiang R Ai Z A long noncoding RNAs signature to improve survival prediction in endometrioid endometrial cancer. J Cell Biochem. (2019) 120:8300–10. 10.1002/jcb.28113 30548294

[31] Wang Y Ren F Chen P Liu S Song Z Ma X. Identification of a six-gene signature with prognostic value for patients with endometrial carcinoma. Cancer Med. (2018) 7:5632–42. 10.1002/cam4.1806 30306731 PMC6247034

[32] Ying J Wang Q Xu T Lyu J. Establishment of a nine-gene prognostic model for predicting overall survival of patients with endometrial carcinoma. Cancer Med. (2018) 7:2601–11. 10.1002/cam4.1498 29665298 PMC6010780

[33] Scolz M Widlund PO Piazza S Bublik DR Reber S Peche LY. GTSE1 is a microtubule plus-end tracking protein that regulates EB1-dependent cell migration. PLoS ONE. (2012) 7:e51259. 10.1371/journal.pone.0051259 23236459 PMC3517537

[34] Subhash VV Tan SH Tan WL Yeo MS Xie C Wong FY. GTSE1 expression represses apoptotic signaling and confers cisplatin resistance in gastric cancer cells. BMC Cancer. (2015) 15:550. 10.1186/s12885-015-1550-0 26209226 PMC4514980

[35] Stelitano D Peche LY Dalla E Monte M Piazza S Schneider C. GTSE1: a novel TEAD4-E2F1 target gene involved in cell protrusions formation in triple-negative breast cancer cell models. Oncotarget. (2017) 8:67422–38. 10.18632/oncotarget.18691 28978043 PMC5620183

[36] Gu Y Lu L Wu L Chen H Zhu W He Y. Identification of prognostic genes in kidney renal clear cell carcinoma by RNAseq data analysis. Mol Med Rep. (2017) 15:1661–7. 10.3892/mmr.2017.6194 28260099 PMC5364979

[37] Zhao P Meng Q Liu LZ You YP Liu N Jiang BH. Regulation of survivin by PI3K/Akt/p70S6K1 pathway. Biochem Biophys Res Commun. (2010) 395:219–24. 10.1016/j.bbrc.2010.03.165 20361940

[38] Chuwa AH Sone K Oda K Ikeda Y Fukuda T Wada-Hiraike O. Significance of survivin as a prognostic factor and a therapeutic target in endometrial cancer. Gynecol Oncol. (2016) 141:564–9. 10.1016/j.ygyno.2016.04.003 27079211

[39] Guo M Lu S Huang H Wang Y Yang MQ Yang Y. Increased AURKA promotes cell proliferation and predicts poor prognosis in bladder cancer. BMC Syst Biol. (2018) 12 (Suppl. 7):118. 10.1186/s12918-018-0634-2 30547784 PMC6293497

[40] Gao X Chen Y Chen M Wang S Wen X Zhang S. Identification of key candidate genes and biological pathways in bladder cancer. PeerJ. (2018) 6:e6036. 10.7717/peerj.6036 30533316 PMC6284430

[41] Zhang MY Liu XX Li H Li R Liu X Qu YQ. Elevated mRNA Levels of AURKA, CDC20 and TPX2 are associated with poor prognosis of smoking related lung adenocarcinoma using bioinformatics analysis. Int J Med Sci. (2018) 15:1676–85. 10.7150/ijms.28728 30588191 PMC6299412

[42] Sun C Cheng X Wang C Wang X Xia B Zhang Y. Gene expression profiles analysis identifies a novel two-gene signature to predict overall survival in diffuse large B-cell lymphoma. Biosci Rep. (2019) 39:BSR20181293. 10.1042/BSR20181293 30393234 PMC6328866

[43] Chai Y Xue H Wu Y Du X Zhang Z Zhang Y. MicroRNA-216b-3p inhibits lung adenocarcinoma cell growth via regulating PDZ binding kinase/T-LAK-cell-originated protein kinase. Exp Ther Med. (2018) 15:4822–8. 10.3892/etm.2018.6020 29805502 PMC5952093

[44] Xiao Y Feng M Ran H Han X Li X. Identification of key differentially expressed genes associated with nonsmall cell lung cancer by bioinformatics analyses. Mol Med Rep. (2018) 17:6379–86. 10.3892/mmr.2018.8726 29532892 PMC5928621

[45] Lee JH Kim MS Yoo NJ Lee SH. Absence of KNSTRN mutation, a cutaneous squamous carcinoma-specific mutation, in other solid tumors and leukemias. Pathol Oncol Res. (2016) 22:227–8. 10.1007/s12253-015-9993-9 26433880

[46] Jaju PD Nguyen CB Mah AM Atwood SX Li J Zia A. Mutations in the kinetochore gene KNSTRN in basal cell carcinoma. J Invest Dermatol. (2015) 135:3197–200. 10.1038/jid.2015.339 26348826 PMC4747638

