# Supplementary material for: Clinical and Expression Significance of AKT1 by Co-expression Network Analysis in Endometrial Cancer
Source: Front Oncol. 2019 Nov 6;9:1147. doi: 10.3389/fonc.2019.01147 (PMC6852383; doi:10.3389/fonc.2019.01147)
Supplement: Supplementary file 1 [file Data_Sheet_1.zip › Legends.DOCX]

**Supplementary Legends**

**Supplementary S1.** The results of GSEA analysis among different treatment groups.

**Supplementary S2.** The results of DEGs among different groups.

**Supplementary S3.** The results of DEGs among different groups.

**Supplementary S4.** The results of KEGG pathway enrichment.

**Supplementary S5.** The results of KEGG pathway enrichment analysis.

**Supplementary S6.** Genes in each module by WGCNA method.

**Supplementary S7.** The enrichment analysis of genes in brown and yellow module.

**Supplementary S8.** The enrichment analysis of genes in brown and yellow modules. (A) The enrichment network of genes in brown module. (B) The enrichment network of genes in yellow module.

**Supplementary S9.** Characteristics of endometrial cancer patients.
