# Supplementary material for: Clinical and Expression Significance of AKT1 by Co-expression Network Analysis in Endometrial Cancer
Source: Front Oncol. 2019 Nov 6;9:1147. doi: 10.3389/fonc.2019.01147 (PMC6852383; doi:10.3389/fonc.2019.01147)
Supplement: Supplementary file 1 [file Data_Sheet_1.zip › Supplementary File 9.DOCX]

**Supplementary S9.** Characteristics of endometrial cancer patients.

| **Characteristic** | **No.** | **%** |
| --- | --- | --- |
| **Age (y), median (range)** | 58(36-76) | 100% |
| **Stage** |  |  |
| III | 81 | 75.7% |
| IV | 26 | 24.3% |
| **Histological subtype** |  |  |
| Endometroid | 100 | 100% |
| **Differentiation grade** |  |  |
| G1 | 14 | 13.1% |
| G2 | 46 | 43.0% |
| G3 | 30 | 28.0% |
| Unknown | 17 | 15.9% |
| **Residual disease** |  |  |
| optimal | 87 | 81.3% |
| suboptimal | 12 | 11.2% |
| unknown | 8 | 7.5% |
