# Supplementary figures and images for: Clinical and Expression Significance of AKT1 by Co-expression Network Analysis in Endometrial Cancer
Source: Front Oncol. 2019 Nov 6;9:1147. doi: 10.3389/fonc.2019.01147 (PMC6852383; doi:10.3389/fonc.2019.01147)

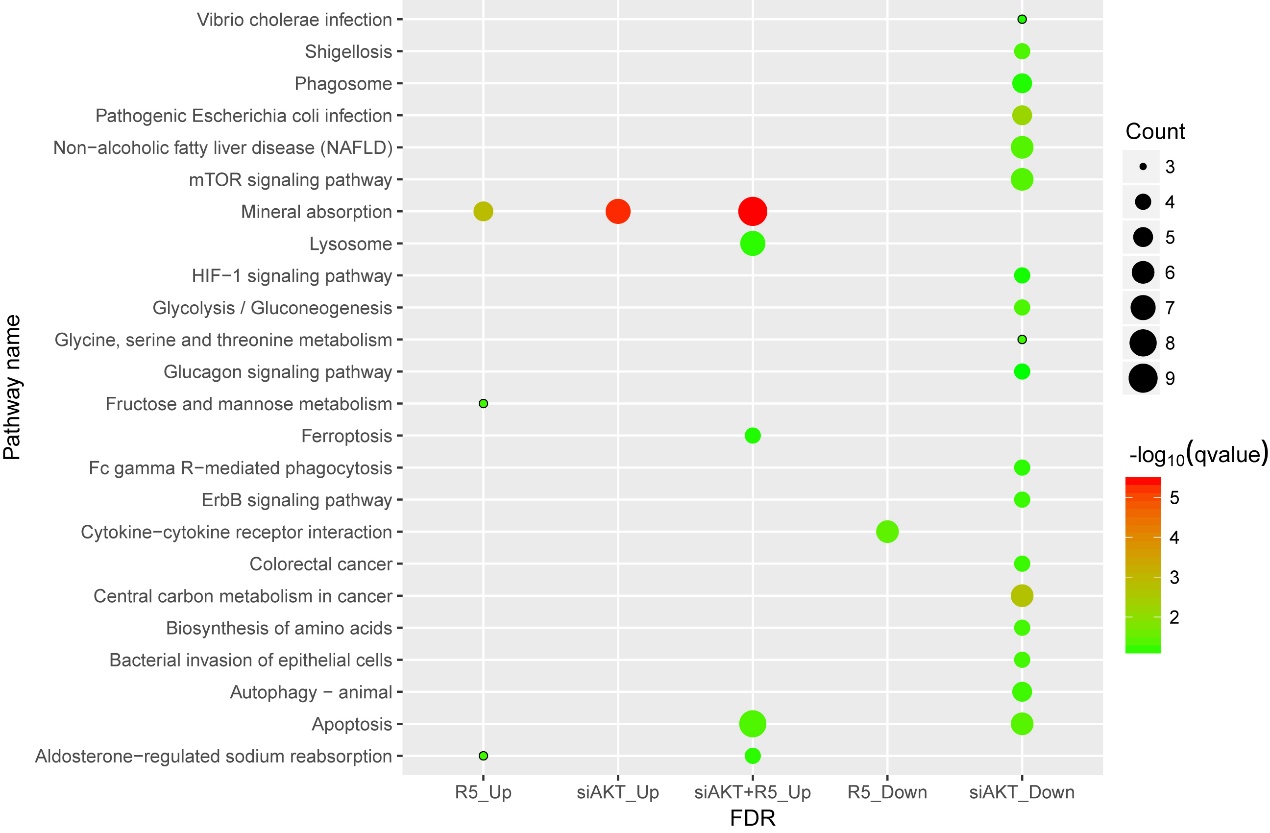

Supplement: Supplementary file 1 [file Data_Sheet_1.zip › Supplementary File 5.DOCX]

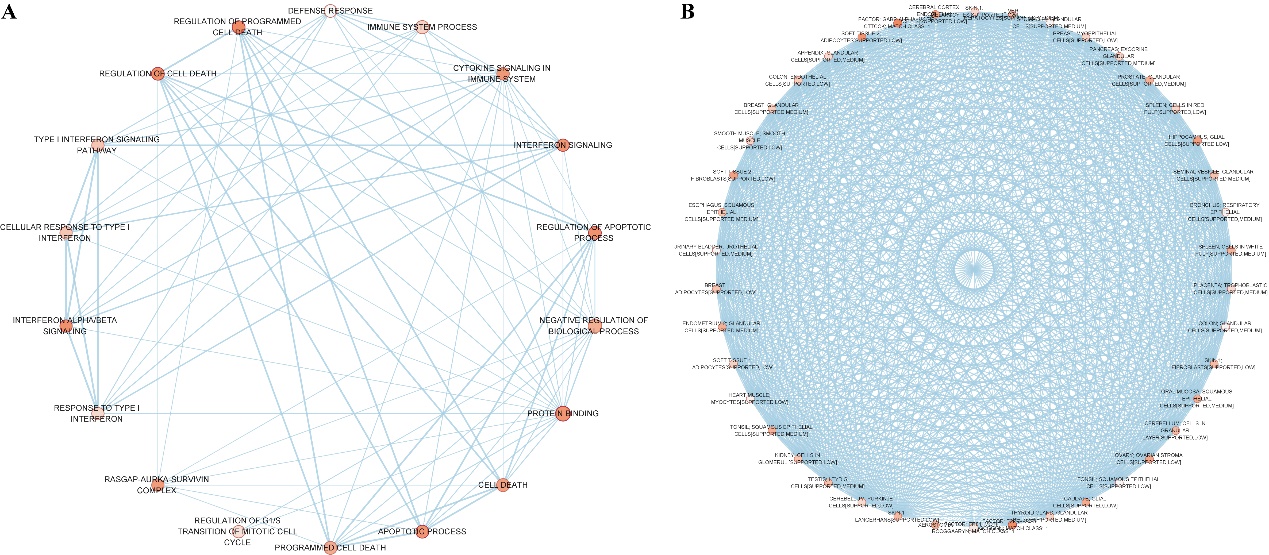

Supplement: Supplementary file 1 [file Data_Sheet_1.zip › Supplementary File 8.DOCX]
